# Application Effects of Single-Lumen Endotracheal Tube Intubation for General Anesthesia in Totally Thoracoscopic Cardiac Surgery

**DOI:** 10.21470/1678-9741-2024-0339

**Published:** 2025-08-22

**Authors:** Xuemei Yi, Lei Wang

**Affiliations:** 1 Second Operating Room, The First Hospital of Jilin University, Changchun, Jilin, People’s Republic of China; 2 Department of Cardiac Surgery, The First Hospital of Jilin University, Changchun, Jilin, People’s Republic of China

**Keywords:** Aorta, Thoracic, Thoracic Diseases, Extracorporeal Circulation, Oxygen Saturation, Recovery Room, Anesthesia, Inhalation, Resuscitation, Intubation.

## Abstract

**Introduction:**

The rapid advancement of medical technology has enabled the application of single-lumen endotracheal tube (SLET) incubation anesthesia in thoracoscopic surgeries for thoracic diseases, demonstrating promising results. This study aims to explore the application of extracorporeal circulation (ECC) and combined intravenous-inhalation anesthesia (CIIA) with SLET intubation in totally thoracoscopic cardiac surgery (TTCS).

**Methods:**

In this single-center, double-blind, randomized controlled trial, we assessed primary outcomes, including intraoperative metrics and postoperative conditions. Secondary outcomes included the number of patients achieving spontaneous resuscitation and those requiring extracorporeal defibrillation after opening the ascending aorta, alertness/sedation scores five minutes post-extubation, and incidence of postoperative complications.

**Results:**

The observation group showed shorter durations in the anesthesia recovery room, intensive care unit retention, extubation, eye-opening time, and postoperative hospital stay compared to the control group (t = 5.913, 8.820, 7.792, 6.904, 11.140; all P < 0.001) and had higher proportion of patients with an alertness/sedation score of five (43/109, 39.45%) and rate of spontaneous resuscitation after opening the ascending aorta (97/109, 88.99%) compared to the control group ([8/109, 34%], [84/109, 77.06%]). In contrast, the rate of external electrical defibrillation (12/109, 11.01%) and the incidence of postoperative complications (2/109, 1.83%) were lower than in the control group ([25/109, 22.94%], [10/109, 9.17%]) (χ^^2^^ = 31.350, 5.501, 5.644; all P < 0.05).

**Conclusion:**

Maintaining oxygen saturation in thoracoscopic surgery requires effective cooperation of anesthesia and ECC. The combined use of ECC and CIIA with SLET intubation in TTCS is a safe, effective approach that warrants broader clinical application.

## INTRODUCTION

**Table:** 

Abbreviations, Acronyms & Symbols	
ASA	= American Society of Anesthesiologists
CIIA	= Combined intravenous-inhalation anesthesia
ECC	= Extracorporeal circulation
ICU	= Intensive care unit
SLET	= Single-lumen endotracheal tube
TTCS	= Totally thoracoscopic cardiac surgery

General anesthesia, which induces an unconscious state via anesthetic agents, acts on neurons to suppress overall brain neuronal activity^[1]^. It is commonly utilized in a variety of clinical settings due to its capability to induce consciousness loss^[2]^. Typically, general anesthesia is achieved using a combination of inhaled anesthetic gases and intravenously administered drugs^[3]^. The use of inhaled anesthetics for both the induction and maintenance of general anesthesia spans over 150 years^[4]^. Besides, when the maintenance of general anesthesia relies solely on intravenous infusion, it is referred to as total intravenous anesthesia^[5]^. This study focuses on evaluating the application effects of extracorporeal circulation (ECC) and combined intravenous-inhalation anesthesia (CIIA) using single-lumen endotracheal tube (SLET) intubation in patients undergoing totally thoracoscopic cardiac surgery (TTCS).

Our literature review identified extensive research on TTCS and ECC applications. For instance, ECC has increasingly been utilized for mechanical support in cases of cardiocirculatory failure^[6]^ and remains a critical component of cardiac surgery^[7]^; pulsatile ECC may enhance the perfusion of vital organs during cardiac procedures^[8]^; and ECC techniques, such as extracorporeal membrane oxygenation and cardiopulmonary bypass, can temporarily assume the function of one or more organs, allowing clinicians time to address underlying pathophysiological conditions^[9]^. Research has also explored the application of SLET intubation in cardiac surgery, including its role in two-lung ventilation to reduce anesthesia and surgical duration^[10]^. Additionally, SLET intubation without lung isolation has been shown to be a feasible, safe alternative for airway management in robotic cardiac procedures, resulting in shorter hospital stays^[11]^. Inhalation anesthetics continue to play a role in inducing and maintaining anesthesia in the operation room^[12]^. Studies have demonstrated that inhalation anesthesia can help preserve intraoperative cardiac function and reduce postoperative pulmonary complications in thoracic surgery involving one-lung ventilation^[13]^. In heart valve surgeries, no significant differences in survival rates or major postoperative complications were observed between inhalation anesthesia and total intravenous anesthesia^[14]^. However, despite the valuable insights provided by these studies, there remains a gap in the literature regarding the specific application effects of combining ECC with SLET and CIIA in TTCS.

This study aims to explore the specific application effects of ECC combined with SLET and CIIA in TTCS, with a focus on intraoperative factors (such as anesthesia and surgical duration), postoperative conditions (recovery room time, postoperative hospital stay), and complication occurrences. By contributing to a clearer understanding of these application effects, we aim to support more effective anesthesia management strategies in clinical practice, ultimately enhancing surgical safety and improving patient quality of life.

## METHODS

### Ethics Statement

The study was approved by the Ethics Committee of our hospital. All participants provided written informed consent, and the study adhered to the principles outlined in the Declaration of Helsinki.

### Study Design

This single-center, double-blind, randomized, controlled trial was conducted at our hospital and was prospectively registered in the Chinese Clinical Trial Registry (registration number: ChiCTR1800012588; date: January 14, 2018).

### Study Subjects

After obtaining informed consent, 218 patients who were admitted to our hospital between January 2018 and December 2021 and were diagnosed as requiring thoracoscopic cardiac surgery were enrolled in the study. Inclusion criteria were as follows: (1) diagnosis of heart disease; (2) indication for surgical treatment; (3) first-time cardiac surgery; and (4) American Society of Anesthesiologists (ASA) classification I-III. Exclusion criteria were as follows: (1) history of right-sided thoracic surgery or severe adhesion; (2) concurrent severe cerebrovascular or respiratory disease; (3) long-term analgesic, opioids, or corticosteroid use; (4) history of psychiatric illness; (5) inability to remove the tracheal tube early due to hemorrhage, hematoma, or poor systemic condition; and (6) allergy to the study drug.

### Randomization and Blinding

Patients were randomly assigned to the observation group or control group using computer-generated random numbers. Randomization codes were prepared, sealed in sequentially numbered, opaque envelopes, and provided to the researchers for intervention allocation. Apart from the anesthesiologists, all patients, surgeons, nurses, and outcome assessors were blinded to the group assignments.

### Anesthesia Methods

Upon entering the operating room, all patients underwent monitoring of pulse oxygen saturation, electrocardiogram, and invasive blood pressure via the left radial artery. Patients received an intramuscular injection of 0.3 mg scopolamine (Sui Cheng Pharmaceutical Co., Ltd., Henan, China; State Drug Administration: H41021048; specification: 1 mL/0.3 mg) and 1 mg/kg pethidine (Qinghai Pharmaceutical Co., Ltd., Qinghai, China; State Drug Administration: H63020021; specification: 1 mL/50 mg), at 30 minutes before surgery. Anesthesia induction included 0.05 mg/kg imipramine (Jiangsu Jiu Xu Pharmaceutical Co., Ltd., Jiangsu, China; State Drug Administration: H20113433; specification: 1 mL/5 mg), 5 µg/kg fentanyl (Yichang Humanwell Pharmaceutical Co., Ltd., Hubei, China; State Drug Administration: H20003688; specification: 2 mL/0.1 mg), 1 mg/kg propofol (Beijing Fresenius Kabi Pharmaceutical Co., Ltd., Beijing, China; State Drug Administration: HJ20150655; specification: 20 mL/0.2 g), and 0.2 mg/kg cisatracurium (Jiangsu Heng Rui Pharmaceutical Co., Ltd., Jiangsu, China; State Drug Administration: H20060868; specification: 5 mL/10 mg). Mechanical ventilation was initiated five minutes later with left-sided double-lumen bronchial intubation. ECC was performed via the right internal jugular and right femoral veins for drainage and the right femoral artery for perfusion. Thoracoscopic surgery was conducted using left one-lung ventilation and a right thoracic approach before ECC initiation. In the control group, anesthesia maintenance consisted of intermittent boluses of propofol (3 - 5 mg·kg-1·min-1), high-dose fentanyl (30 - 50 µg/kg in total), and cisatracurium. In the observation group, CIIA with SLET intubation was used. Patients were positioned in the supine position. Anesthesia was induced with intravenous benzenesulfonyl cisatracurium (0.2 mg/kg) and propofol (0.5 - 1.0 mg/kg), followed by small tidal volume, high-frequency, pure oxygen positive-pressure ventilation, and early application of pre-parallel procedures, hypothermia, and low flow. Anesthesia maintenance in the observation group included sevoflurane inhalation and intermittent intravenous infusions of 2 mg cisatracurium and 25 - 30 µg propofol.

### Outcome Measures

Primary outcomes included surgical conditions (duration of surgery, ECC, myocardial hemodynamic blockade, anesthesia) and postoperative conditions (duration in anesthesia recovery room, intensive care unit [ICU] retention, extubation, eye-opening time, and length of postoperative hospital stay). Secondary outcomes included the number of patients achieving spontaneous resuscitation and extracorporeal defibrillation after opening the ascending aorta, alertness/sedation scores^[15]^ five minutes after extubation, and postoperative complications (such as secondary thoracoscopic exploration for hemostasis, pulmonary atelectasis, recurrent malignant ventricular arrhythmias, and severe acute lung injury). The alertness/sedation score criteria were as follows: five points for rapid response to a normal tone of voice; four points for slow response to a normal tone of voice; three points for response to repeated or loud calls; two points for response to tapping or shaking; one point for no response.

### Statistics

Data analysis was conducted using IBM Corp. Released 2011, IBM SPSS Statistics for Windows, version 20.0, Armonk, NY: IBM Corp. Categorical data were presented as (case [%]), with comparisons made using the χ^^2^^ test or Fisher’s exact test. The Shapiro-Wilk test was applied to assess data normality. Normally distributed continuous data were presented as means ± standard error of the mean/standard deviation, and comparisons between groups were conducted using the *t* -test. *P* < 0.05 was considered statistically significant.

## RESULTS

### General Data

**Fig. 1:**
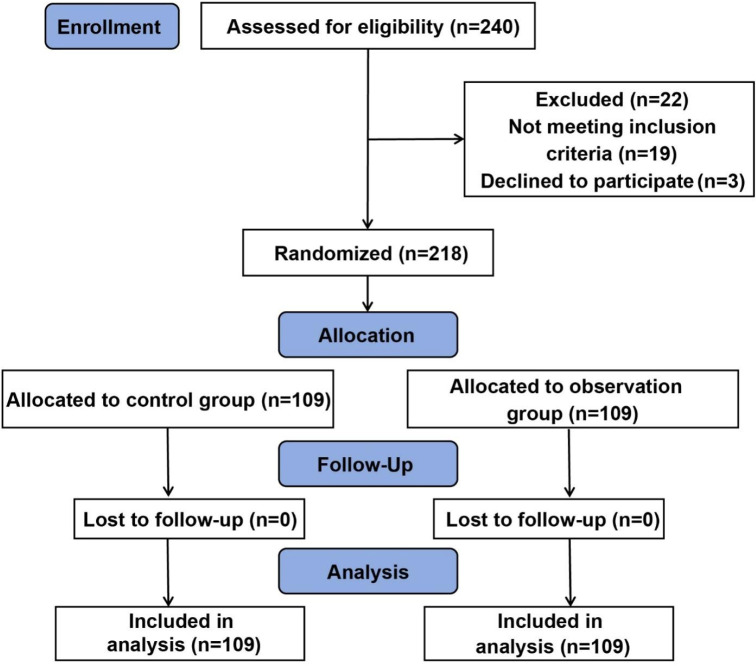
Research objects are included in the flow chart.

Figure 1 presented the consolidated standards of reporting trials flowchart for this study, which included 240 patients recruited between January 2018 and December 2021. A total of 22 patients were excluded due to either not meeting the inclusion criteria or declining to participate. Ultimately, 218 patients successfully completed the study protocol and were randomly assigned to either the control group or the observation group. There were no significant differences in age, sex, ASA classification, or type of surgery between the two groups, indicating baseline comparability ( *P* > 0.05) (Table 1).

**Table 1:** General data between the two groups.

Indicators	Control group (n = 109)	Observation group (n = 109)	t-value/χ^^2^^ value	*P* -value
Age (years)	30.49 ± 3.07	30.97 ± 3.27	1.474	0.142
Sex (cases)			0.486	0.486
Male	44	39		
Female	65	70		
ASA classification (cases)			5.141	0.077
I	39	46		
II	61	46		
III	9	17		
Surgery types (cases)			2.707	0.439
Atrial septal defect repair	61	52		
Ventricular septal defect repair	8	12		
Mitral valve replacement	32	32		
Mitral valvuloplasty	8	13		

ASA=American Society of Anesthesiologists

### Surgery and Postoperative Conditions

No significant differences were observed between the two groups in anesthesia duration, surgery duration, ECC time, or myocardial hemodynamic blockade time ( *P* > 0.05). However, the observation group showed shorter times for anesthesia recovery room stay, ICU retention, eye-opening, and postoperative hospital stay compared to the control group ( *P* < 0.05) (Table 2).

**Table 2:** Surgery conditions and postoperative conditions between the two groups.

Indicators	Control group (n = 109)	Observation group (n = 109)	*t* -value	*P* -value
Anesthesia time (min.)	231.25 ± 29.74	229.37 ± 31.56	0.453	0.651
Surgery time (min.)	298.31 ± 27.36	292.98 ± 26.41	1.464	0.145
Extracorporeal circulation time (min.)	152.37 ± 18.64	149.54 ± 15.70	1.212	0.227
Myocardial blood supply blocking time (min.)	75.96 ± 12.35	73.35 ± 11.10	1.641	0.102
Anesthesia recovery room time (h)	6.77 ± 1.87	5.48 ± 1.10	5.913	< 0.001
Intensive care unit retention time (days)	3.20 ± 0.73	2.39 ± 0.62	8.82	< 0.001
Extubation time (min.)	23.13 ± 5.59	17.82 ± 4.40	7.792	< 0.001
Eye-opening time (min.)	22.71 ± 7.96	16.25 ± 5.66	6.904	< 0.001
Postoperative hospital stay (days)	10.17 ± 1.55	8.13 ± 1.12	11.14	< 0.001

### Alertness/Sedation Scores

The percentage of patients achieving a five-point alertness/sedation score in the observation group was 39.45%, which was significantly higher than the 7.34% in the control group ( *P* < 0.05) (Table 3).

**Table 3:** Alertness/sedation scores between the two groups, n (%).

Grouping	n	5 points	4 points	3 points	2 points	1 point	*P* -value
Control group	109	8 (7.34)	34 (31.19)	56 (51.38)	8 (7.34)	3 (2.75)	< 0.001
Observation group	109	43 (39.45)	56 (51.38)	8 (7.34)	1 (0.92)	1 (0.92)	

### Heart Resuscitation

The rate of spontaneous resuscitation after opening the ascending aorta in the observation group was 88.99%, higher than the 77.06% in the control group. Additionally, the rate of resuscitation after external defibrillation was lower in the observation group (11.01%) compared to the control group (22.94%) ( *P* < 0.05) (Table 4).

**Table 4:** Heart resuscitation between the two groups, n (%).

Group	n	Automatic resuscitation rate after opening the ascending aorta	Resuscitation rate after external defibrillation	*P* -value
Control group	109	84 (77.06)	25 (22.94)	0.029
Observation group	109	97 (88.99)	12 (11.01)	

### Complications

The complication rate in the observation group was 1.83%, significantly lower than the 9.17% observed in the control group ( *P* < 0.05) (Table 5).

**Table 5:** Complications between the two groups, n (%).

Group	n	Secondary thoracoscopic exploration for hemostasis	Pulmonary atelectasis	Recurrent malignant ventricular arrhythmias	Severe acute lung injury	Overall complications	*P* -value
Control group	109	5 (4.59)	3 (2.75)	1 (0.92)	1 (0.92)	10 (9.17)	0.033
Observation group	109	1 (0.92)	1 (0.92)	0	0	2 (1.83)	

## DISCUSSION

The management of anesthesia in TTCS has been widely debated and researched^[16]^. This study focused on the clinical value of ECC combined with CIIA and SLET intubation in patients undergoing TTCS.

Previous studies have emphasized the importance of anesthetic agents in improving regional tissue oxygenation, particularly in critical patients and complex surgical procedures^[17]^. Single-lumen tube intubation has been associated with reduced mean ventilation time, ICU stay, and hospital stay^[11]^. In our study, we compared intraoperative and postoperative outcomes between the two groups, finding that the observation group had significantly shorter anesthesia recovery room stay, ICU retention, extubation, eye-opening time, and postoperative hospital stay compared to the control group. Additionally, no significant differences were found between the groups in anesthesia time, surgery time, ECC time, or myocardial hemodynamic blockade time.

In assessing alertness/sedation scores, the observation group exhibited a higher proportion of patients achieving a five-point alertness/sedation score (39.45%) compared to the control group (7.34%). Regarding heart resuscitation, the observation group had a higher spontaneous resuscitation rate after opening the ascending aorta (88.99%) compared to the control group (77.06%) and a lower need for resuscitation via external defibrillation (11.01% *vs.* 22.94%). These findings align with previous studies suggesting a higher success rate of cardiovascular surgery when utilizing ECC^[18]^.

Other studies indicate that presternotomy ECC can lower the risk of adverse events in patients undergoing resternotomy. Routine implementation of presternotomy ECC has been shown to lower the risk of damage to mediastinal structures during reentry and to facilitate easier repair^[19]^. In our study, the incidence of complications in the observation group (1.83%) was significantly lower than in the control group (9.17%).

### Limitations

This study’s limitations include a lack of sample size calculations and limited clinical data. Despite the promising results, there remain areas for improvement. Future studies should be focused on optimizing ECC protocols and evaluating the effects of varying ECC approaches on patients’ vital signs and postoperative recovery to enhance evidence for clinical decision-making. Additionally, conducting long-term follow-ups to assess postoperative cardiac function, quality of life, and other indicators will provide a more comprehensive basis for clinical applications.

## CONCLUSION

In conclusion, this study demonstrates that oxygen saturation maintenance in thoracoscopic surgery requires close cooperation between anesthesia and ECC. Using SLET intubation, an ECC protocol involving pre-parallel early turnover, early hypothermia, low flow, followed by post-parallel late respiratory medication, and delayed blood return proved to be safe and effective. The findings have clinical significance: in TTCS, the close cooperation of ECC, CIIA, and SLET intubation effectively maintains patients' blood oxygen saturation, reduces surgical risks, and minimizes complication rates. Furthermore, our study proposes an optimized ECC method that incorporates early turning, early hypothermia, and low flow at initial stage, followed by delayed respiratory medication administration and blood return, which may contribute to reduced surgical trauma and shorter recovery times. This study provides a foundation for further exploration of ECC and CIIA with SLET intubation in TTCS patients.
